# GCN2 Deficiency Enhances Protective Effects of Exercise on Hepatic Steatosis

**DOI:** 10.1155/2020/1454396

**Published:** 2020-11-24

**Authors:** Xueting Luo, Xiaowei Shi, Zhongguang Sun, Jing Xiao, Hui Song, Guo Lu, Xin Xu

**Affiliations:** ^1^School of Kinesiology, Shanghai University of Sport, Shanghai 200438, China; ^2^School of Physical Education, East China University of Technology, Nanchang, 330013 Jiangxi, China

## Abstract

**Background:**

Combined aerobic and resistance training has been demonstrated to benefit glycemic control and reverse nonalcoholic fatty liver disease in childhood obesity. General control nonderepressible 2 (GCN2) deficiency has been reported to attenuate hepatic steatosis and insulin resistance. However, whether GCN2 impacts the positive effects of combined aerobic and resistance exercise remains unknown.

**Objectives:**

To investigate whether combined aerobic and resistance exercise improves hepatic steatosis and glucose intolerance and the role GCN2 plays in mediating the metabolic regulation of exercise.

**Methods:**

Wild-type (WT) and *GCN2* knockout (GCN2KO) mice were fed a high-fat diet (HFD) for 25 weeks. The WT and GCN2KO mice performed exercise (treadmill running + ladder climbing) during the last eight weeks. Their body and liver weights, their triglyceride content, and their levels of aspartate transaminase (AST), alanine transaminase (ALT), and blood glucose were measured, and the expressions of proteins involved in the GCN2/eIF2*α*/ATF4 pathway and the glucolipid metabolism-related proteins (e.g., p-AMPK, SIRT1, PPAR*α*, PGC-1*α*, GLUT4, and p-GSK-3*β*) were determined.

**Results:**

The body weight of WT and GCN2KO mice continued to increase until the end of the experiment. The liver weights, hepatic triglyceride content, and AST and ALT levels of the exercised mice were significantly reduced compared to those of the sedentary mice. Exercise improved blood glucose levels and glucose clearance ability in the WT mice, but the glucose intolerance of GCN2KO mice was not improved. Exercise increased PGC-1*α*, GLUT4, and p-GSK-3*β* expressions in the WT rather than the GCN2KO mice. Interestingly however, exercise-trained GCN2KO mice were better protected against hepatic steatosis with downregulated expressions of p-eIF2*α* and ATF4, upregulated expressions of p-AMPK and SIRT1, and the presence of PPAR*α* in the liver, compared to the exercised WT mice.

**Conclusion:**

Combined aerobic and resistance exercise had positive effects on hepatic steatosis and the control of glucose intolerance. GCN2 was found to be necessary for exercise-induced improved glucose intolerance. However, the better efficacy in improving hepatic steatosis by exercise in the *GCN2*-deficient mice enhanced liver lipid metabolism, at least partially, via the AMPK/SIRT1/PPAR*α* pathway.

## 1. Introduction

Obesity is one of the main factors that induce various chronic metabolic diseases, including nonalcoholic fatty liver disease (NAFLD), hyperglycemia, dyslipidemia, and type 2 diabetes mellitus (T2DM) [1]. NAFLD is the most common chronic liver disorder, which is more common in patients with T2DM [2]. Lifestyle interventions such as exercise are considered the primary method to prevent diabetes mellitus and NAFLD [3, 4]. Evidence suggests that both aerobic exercise and combined aerobic and resistance exercise improve metabolic syndrome in T2DM [5]. Several studies have demonstrated that the greatest improvements in glycemic control were achieved with combined aerobic and resistance training [6, 7]. Evidence also suggests that combined aerobic and resistance training effectively attenuated juvenile obesity-related NAFLD [8], which is associated with modified hepatic lipid-metabolism-related gene induced by high-fat diet (HFD). However, few studies have focused on the effect of combined aerobic and resistance training on NAFLD in adults and liver tissue-specific mechanisms.

General control nonderepressible 2 kinase (GCN2) is an amino acid (AA) sensor found in various cells, from yeast to mammalian cells [9]. In mammals, GCN2 is expressed in a variety of tissues but most significantly in the liver and brain [10]. Upon activation by limited dietary protein, GCN2 phosphorylates eukaryotic initiation factor 2 (eIF2*α*), represses the translation of global mRNA, and then increases the translation of activating transcription factor 4 (ATF4) mRNAs to encode proteins, which is required for the cells to recover from the initial insult [11]. Recent studies revealed that the *GCN2* gene was required for repressing liver triglyceride synthesis during a period of leucine-deprived diet [12] and that leucine deprivation increased insulin sensitivity by activating GCN2 and AMPK [13]. Nevertheless, the deletion of brain-specific *GCN2* caused defective triglyceride storage in the liver of mice when dietary fat in perinatal nutrition was increased [14]. Another study also reported that the knockout of *GCN2* attenuated hepatic steatosis and insulin resistance in mice fed with HFD for 12 weeks [15]. To date, whether the exercise-induced improvements in hepatic steatosis and insulin resistance of mice were mediated via GCN2 remains to be reported and understood.

The present study was conducted firstly to investigate whether combined aerobic and resistance exercise had beneficial effects on reversing hepatic steatosis and glucose intolerance. Various parameters including body and liver weights, triglyceride content, and glucose intolerance were measured in wild-type (WT) and *GCN2* knockout (GCN2KO) mice. Secondly, the role of GCN2 in exercise-induced metabolic adaptations in fatty livers was explored. This study proposed the interesting hypothesis that combined aerobic and resistance exercise produced integrated improvements in hepatic steatosis and glucose intolerance by reducing hepatic GCN2. Although the beneficial effects of exercise on glucose tolerance appear to be GCN2-dependent, the present research found that exercise could induce liver weight loss, and/or the improvement of hepatic steatosis, which were only partly dependent on GCN2 inhibition. Thirdly, it is proposed that the exercise-induced hepatic steatosis improvement in obese mice with GCN2 deficiency was mediated by other upstream eIF2*α* kinases, eIF2*α*/ATF4 signaling, and AMP-activated protein kinase (AMPK)/deacetylase sirtuin-1 (SIRT1)/peroxisome proliferator-activated receptor (PPAR) alpha pathway in the liver.

## 2. Materials and Methods

### 2.1. Animals

All animal experiments were conducted with permission from the Institutional Animal Care and Use Committee at the Shanghai University of Sport. The experiments were performed in accordance with the guidelines of the Institutional Animal Care and Use Committee. The GCN2KO mice were purchased from the Jackson Laboratory (Bar Harbor, ME, USA) and were then crossed with the C57BL/6J strain (obtained from the Shanghai Laboratory Animal Center (SLAC), Chinese Academy of Sciences, Shanghai, China). Six- to eight-week-old WT male mice and littermate GCN2KO mice were placed in cages under a controlled 12-hour light/12-hour darkness cycle at 22 ± 2°C and 55% relative humidity. All mice were allowed free access to HFD (60% of calories were from fat; FBSH Biomart Co., Shanghai, China) and water throughout the experimental 25-week period, and their body weight was recorded once a week.

### 2.2. HFD Feeding and Exercise Protocol

Mice were randomly divided into four groups (10 mice per group; *n* = 10): (1) sedentary WT HFD-fed mice (WT HFD); (2) exercised WT HFD-fed mice (WT HFD+EXE); (3) sedentary *GCN2* knockout HFD-fed mice (GCN2KO HFD); and (4) exercised *GCN2* knockout HFD-fed mice (GCN2KO HFD+EXE). After 16 weeks, mice in the exercise groups were allowed to acclimatize themselves to the exercises for one week (adaptation period), during which they performed treadmill running (8 m/min, 0° incline, 30 min duration) and ladder climbing (1 m long with 1.5 cm-spaced grids, at an 80° incline, without any weights attached to their tails). After this period, these mice were exposed to combined aerobic and resistance exercises for eight weeks [16]. In terms of aerobic exercise, the mice performed treadmill running at a speed of 10–12 m/min for 60 min at 0° incline [17]. By way of resistance exercise, the mice executed ladder climbing (at a 90° incline) with no tail loading for a total of 4 repetitions/set and 4 sets/day [18]. While exercising, the mice were stimulated by gentle tail touching (rather than electrical stimulation) to climb to the top as fast as they could and were allowed to rest for 1 min between repetitions and 2 min between sets. The mice performed this exercise on alternate days, three days a week.

### 2.3. Liver Tissue Collection and Measurement of Triglyceride (TG) Concentration

The concentration of TG in the liver was measured using a commercial triglyceride quantification assay kit (BioVision, USA). Briefly, 0.1 g of liver tissue from each mouse was homogenized in chloroform/methanol (2 : 1*v*/*v*) by using a Polytron homogenizer. Lipid extracts were prepared by the Folch method. Extraction of lipids was dissolved in isopropanol and then subjected to TG content measurement according to the manufacturer's instructions. The TG concentrations were measured using a microplate reader (BioTek, USA) and were normalized and expressed as milligrams per gram of tissue.

### 2.4. Liver Function and Glucose Tolerance Test

Serum was separated from whole blood samples after centrifugation. The levels of AST and ALT were detected in serum using an automatic biochemical analyzer (AU480, Beckman Coulter, USA). An intraperitoneal glucose tolerance test (GTT) was conducted on completion of the 25-week experimental period. After mice had undergone a 12-hour overnight fast, they were injected intraperitoneally with a glucose solution of 2 g per 1 kg of body weight. Subsequently, their tail blood glucose levels were measured using blood glucose test strips (OneTouch Ultra, Johnson & Johnson, USA) at 30, 60, 90, and 120 min postinjection.

### 2.5. Western Blot

Protein was extracted from 30 mg of frozen liver tissue. The tissue samples were first homogenized in 200 *μ*l of RIPA buffer, and the homogenate was then centrifuged at 12,000 × g for 20 min at 4°C. The protein concentration in the supernatant was determined using a bicinchoninic acid (BCA) protein assay kit (Beyotime, China). Protein with a concentration of at least 8% was separated by SDS-polyacrylamide gel electrophoresis (SDS-PAGE). The protein was then transferred to a polyvinylidene fluoride (PVDF) membrane (Millipore, Bedford, MA, USA). The membrane was then blocked with nonfat milk powder (5%) in Tris-buffered saline containing 0.1% Tween 20 (TBST) for two hours at room temperature. After the membrane had been washed three times (for 10 min each) with TBST, it was incubated with primary antibodies at 4°C overnight, followed by incubation with conjugated secondary antibody anti-rabbit IgG at room temperature for one hour. The primary antibodies included those against GCN2 (3302), eIF2*α* (9722), phospho-eIF2*α* (9721), ATF4 (11815), *β*-tubulin (2128), AMPK (5831), phosphor-AMPK (Thr172; 2535), GSK3*β* (9315), and phospho-GSK3*β* (Ser9; 9323) which had been obtained from Cell Signaling Technology (Danvers, USA) and those against SIRT1 (ab12193), PPAR*α* (ab24509), and GLUT4 (ab654), which had been purchased from Abcam (Cambridge, USA), in addition to PGC-1*α* (nbp1-04676), bought from Novus Biologicals (USA). The antibodies were detected using an enhanced chemiluminescence (ECL) solution (Millipore Immobilon Western, USA) and visualized by a Fusion FX6-XT system (Vilber Lourmat, France).

### 2.6. Statistical Analysis

All experimental data were presented as means ± a standard error margin of the mean (SEM). Graphical preparation and data analysis were carried out using GraphPad Prism 7 and SPSS 24.0, respectively. The differences between the groups were subjected to Student's *t*-test or two-way ANOVA. For the two-way ANOVA method, the Tukey post hoc analysis was carried out to determine the differences between the groups. Repeated measures ANOVA was performed to measure body weight and GTT data. The threshold for differences to be deemed significant was set at *p* < 0.05.

## 3. Results

### 3.1. Exercise-Mediated Protection against Diet-Induced Liver Weight and Liver TG Content Gain Is Potentiated in GCN2KO Mice

**Figure 1:**
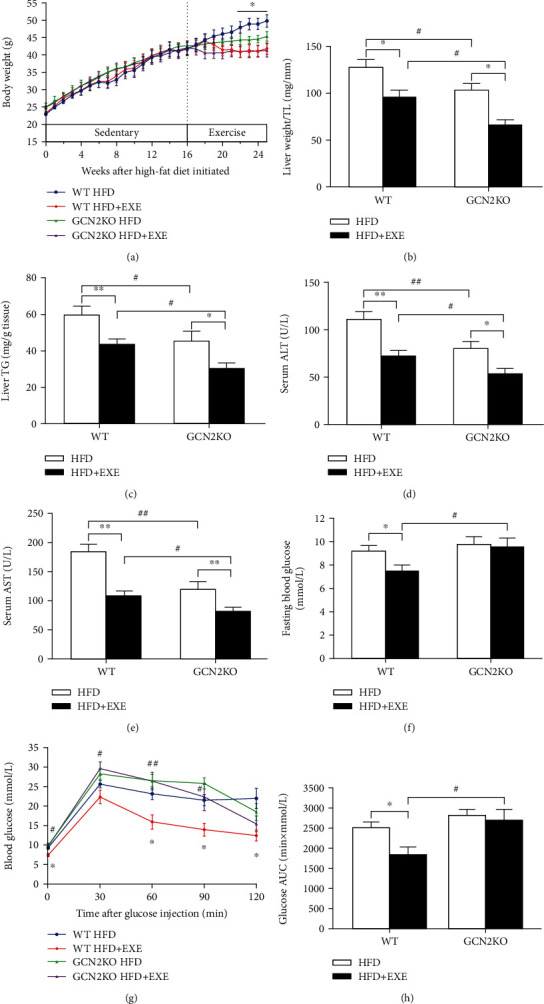
Effects of exercise on body weight, liver weight, liver triglycerides, liver function, and glucose tolerance of mice: (a) changes in body weight (*n* = 8–10); (b) liver weight normalized to the tibia length (TL); (c) liver triglyceride (TG) content (*n* = 8–10); (d) serum ALT levels; (e) serum AST levels (*n* = 8–10); (f) fasting blood glucose concentration; (g) blood glucose concentration; and (h) respective areas under the curve (AUC) (*n* = 6–10). ^∗^*p* < 0.05 and ^∗∗^*p* < 0.01, for sedentary *vs.* exercise difference, as determined by two-way ANOVA or two-way repeated measures ANOVA and Tukey post hoc test. ^#^*p* < 0.05 and ^##^*p* < 0.01 for the difference between WT and GCN2KO, as determined by two-way ANOVA or two-way repeated measures ANOVA and Tukey post hoc test.

The body weights of GCN2KO and WT mice on the HFD differed neither at the beginning nor at the end of the study, while the increase in the former was less than that in the latter. Exercise-induced body weight loss was evident in the WT mice between 22 and 25 weeks. No significant difference in exercise-induced body weight in GCN2KO mice was observed, suggesting that exercise did not result in further weight loss in the GCN2KO mice on the HFD (Figure 1(a)). Interestingly, the liver weights of GCN2KO mice in both HFD and HFD+EXE groups were significantly lower than those of WT mice in the same groups (Figure 1(b)). The same result was observed in respect of liver TG contents (Figure 1(c)), suggesting that GCN2 deficiency had a protective effect against gains in liver weight and liver TG content. However, liver weight and liver TG content decreased similarly with exercise in both genotypes, but exercise prevented liver weight gain and hepatic steatosis more effectively in the GCN2KO mice (Figures 1(b) and 1(c)). Exercise decreased ALT and AST levels in both genotypes compared to the HFD groups, while GCN2 deficiency resulted in significantly reduced levels of serum ALT and AST in GCN2KO compared to WT mice (Figures 1(d) and 1(e)), indicating that exercise markedly improved HFD-induced liver dysfunction in GCN2KO mice.

Furthermore, the fasting blood glucose concentration of the WT HFD+EXE group was significantly lower than that of the WT HFD group (Figure 1(f)), and the glucose clearance ability of WT mice in HFD+EXE group was higher than that of those in the HFD group. In contrast, fasting blood glucose and glucose intolerance levels in the GCN2KO HFD+EXE group remained nearly unchanged compared to the GCN2KO HFD group, indicating that GCN2 deficiency did impair the GCN2KO HFD+EXE group's glucose clearance ability, which exercise failed to ameliorate (Figures 1(g) and 1(h)).

### 3.2. Exercise Generated a Decreased eIF2*α*/ATF4 Pathway in GCN2KO Mice

**Figure 2:**
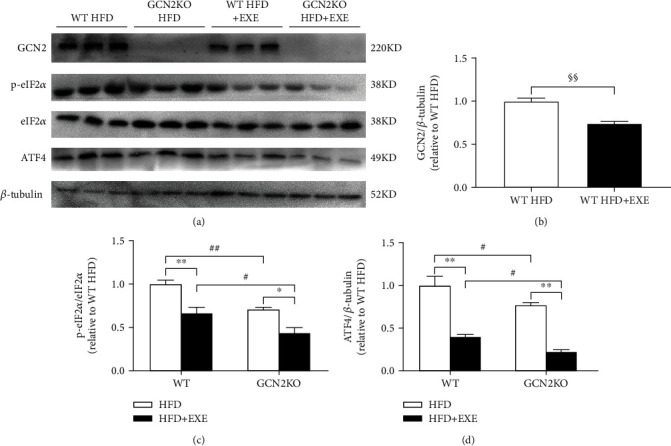
Expression level of proteins involved in the GCN2/eIF2*α*/ATF4 pathway in the liver of mice: (a) representative immunoblot; (b–d) the expression levels of (b) GCN2, (c) phosphorylated eIF2*α*, and (d) ATF4 proteins. Data are means ± SEM (*n* = 6). ^§§^*p* < 0.01 for the difference between sedentary and exercised WT mice groups, as determined by Student's *t*-test. ^∗^*p* < 0.05 and ^∗∗^*p* < 0.01 for the difference between sedentary and exercised regimes, as determined by two-way ANOVA and Tukey post hoc test. ^#^*p* < 0.05 and ^##^*p* < 0.01 for the difference between WT and GCN2KO groups, as determined by two-way ANOVA and Tukey post hoc test.

As shown in Figure 2(a), the expression of GCN2 was not detected in the livers of any GCN2KO mice; the model is thus validated. GCN2 protein level in the livers of WT HFD+EXE mice was significantly reduced by 26% compared to the findings in the WT HFD mice (Figure 2(b)), indicating that exercise reduced the expression of GCN2. Consistent with the antihepatic steatosis effects, exercise also significantly reduced the expressions of phosphorylated eIF2*α* and ATF4 proteins in the WT mice. A decrease in liver eIF2*α* phosphorylation and ATF4 was also found in both the HFD and HFD+EXE groups for GCN2KO mice, but the decrease was more obvious in the WT mice. Moreover, eIF2*α*/ATF4 pathways were further reduced by the exercise regime in the livers of the GCN2KO mice, suggesting a significant exercise-induced downregulation of the eIF2*α*/ATF4 pathway in the GCN2KO mice (Figures 2(c) and 2(d)).

### 3.3. Exercise Regulated Lipid Metabolism through the AMPK/SIRT1/PPAR*α* Pathway in GCN2KO Mice

**Figure 3:**
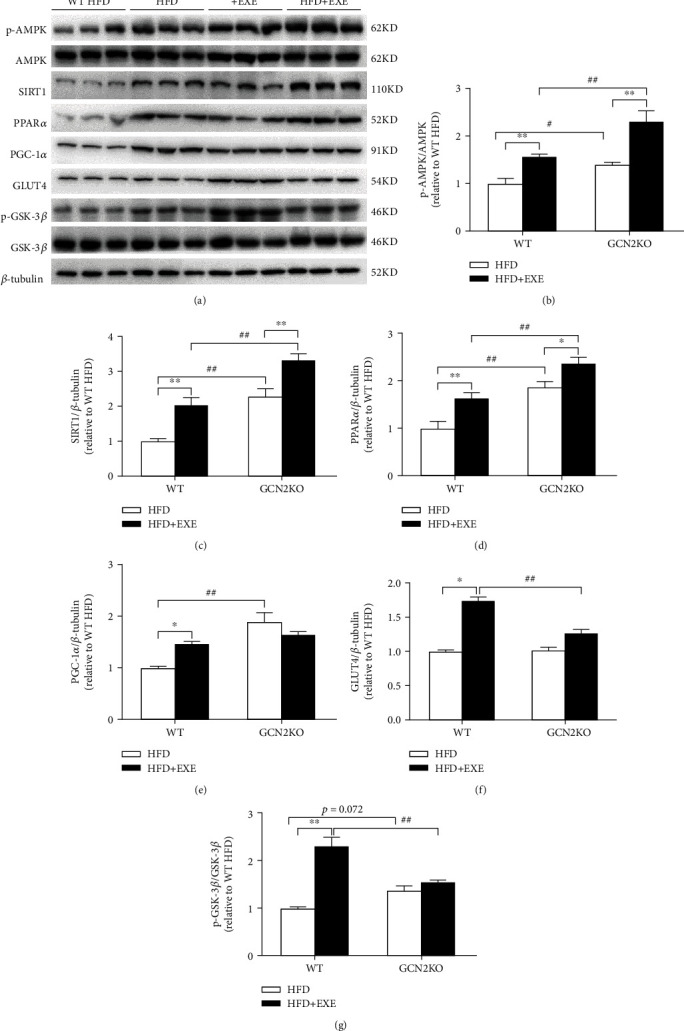
Expression level of proteins involving in the glucolipid metabolic signaling pathway in the liver of mice: (a) representative immunoblot; (b–g) the expression levels of (b) p-AMPK, (c) SIRT1, (d) PPAR*α*, (e) PGC-1*α*, (f) GLUT4, and (g) p-GSK-3*β* proteins. Data are means ± SEM (*n* = 6). ^∗^*p* < 0.05 and ^∗∗^*p* < 0.01 for the difference between sedentary and exercise regimes, as determined by two-way ANOVA and Tukey post hoc test. ^#^*p* < 0.05 and ^##^*p* < 0.01 for the difference between WT and GCN2KO groups, as determined by two-way ANOVA and Tukey post hoc test.

As the glucolipid metabolism was potentially affected by exercise, we assessed the expression of proteins involved in liver glucolipid metabolism-signaling pathway, including phosphorylated AMPK (p-AMPK; at Thr172), SIRT1, PPAR gamma coactivator 1 (PGC1) alpha (PGC-1*α*), PPAR*α*, glucose transporter 4 (GLUT4), and phosphorylated glycogen synthase kinase 3*β* (p-GSK-3*β*; at Ser9). As shown in Figure 3, the expressions of all proteins in the WT HFD + EXE group were significantly increased compared to those in the WT HFD group, indicating that exercise was involved in the control of glucolipid metabolism signaling pathways.

The expressions of p-AMPK, SIRT1, and PPAR*α*, but not PGC-1*α*, GLUT4, and p-GSK-3*β*, in the GCN2KO HFD+EXE group were significantly increased compared to those in the GCN2KO HFD group. When comparing WT HFD and GCN2KO HFD mice, the expressions of p-AMPK, SIRT1, PPAR*α*, and PGC-1*α* in the GCN2KO HFD mice were higher than those in the WT mice, whereas the expressions of GLUT4 and p-GSK-3*β* were nearly identical in each group. The expressions of p-AMPK, SIRT1, and PPAR*α* in the GCN2KO HFD+EXE mice were significantly increased compared to those in the WT HFD+EXE mice, but the expressions of GLUT4 and p-GSK-3*β* were decreased, while those of PGC-1*α* were nearly identical. These results showed that the regulation of glucolipid metabolism signaling pathway through exercise could vary between WT and GCN2KO mice.

## 4. Discussion

This study was conducted to investigate the effects of (combined aerobic and resistance) exercise on hepatic steatosis and glucose intolerance and to understand the role of GCN2 in exercise-induced hepatic glucolipid metabolic adaptations. The GCN2KO mouse model allowed us to explore the specific interactions between exercise and GCN2 deficiency on HFD-induced hepatic steatosis. In this respect, it was observed that (1) combined aerobic and resistance exercise could reduce body and liver weight, liver TG, and serum AST and ALT levels under the HFD condition in WT mice. Although exercise did not affect body weight loss in the GCN2KO genotype, exercise-trained GCN2KO mice were better protected against diet-induced liver weights, liver TG gains, and liver dysfunction compared to exercise-performing WT mice. (2) Combined aerobic and resistance exercise improved fasting glucose and glucose intolerance levels in WT HFD mice, whereas the response of *GCN2*-deficient obese mice to fasting glucose and glucose tolerance was slower and failed to respond favorably to exercise. This result revealed the critical role of GCN2 in exercise-mediated hepatic glucose metabolic adaptations. One surprising observation was that sedentary GCN2KO mice fed HFD for 25 weeks demonstrated poorer glycemic control, which was in contrast to a previous study in which GCN2KO mice fed with HFD for 12 weeks had been found to show attenuated HFD-induced insulin resistance [15]. These distinct studies indicated that both the duration of the HFD and the degree of obesity were key factors in determining responses to glycemic control in GCN2KO mice.

HFD-activated GCN2 phosphorylates eIF2*α*. It is well documented that while phosphorylated eIF2*α* inhibits general translation initiation, it facilitates the translation of ATF4, which is a potential stimulator of lipid biosynthesis and glucose metabolism [19, 20]. How these signaling components mediate the complex responses to exercise formed was thus investigated in the present study. Its findings were that exercise could reduce the expressions of GCN2 in HFD-fed WT mice. *GCN2* deficiency was found to inhibit the expressions of p-eIF2*α* and ATF4, and this inhibitory effect could be enhanced by exercise. Notably, the eIF2*α*/ATF4 pathway was shown to be implicated in the regulation of lipogenesis processes. Thus, the amelioration of hepatic steatosis due to exercise may have partly been related to GCN2 control, although other eIF2*α* kinases may also have contributed to this result. However, in the present study, exercise was found to improve glucose intolerance by decreasing GCN2; paradoxically, GCN2KO mice displayed impaired glucose homeostasis which could not be normalized through exercise. It was proposed that *GCN2* was involved in programming the “thrifty phenotype” and that *GCN2*-deficient leptin receptor-mutated mice displayed a reduced capacity to store triglycerides and an increased susceptibility to developing type 2 diabetes [14]. Significant genotypic differences were observed in the exercised groups, suggesting that the phenotype of glucose metabolic in exercised GCN2KO mice may have been the result of glucose metabolism imbalance that preexisted during exercise. Therefore, the exercise-induced metabolic differences in two mouse genotypes require further investigation.

The beneficial metabolic effects of combined aerobic and resistance exercise on HFD-induced early obesity and NAFLD have been documented in previous research [8]. The modified lipid metabolism produced by combined aerobic and resistance exercise was found to limit intrahepatic lipid accumulation by downregulating the expression of fatty acid synthase, fatty acid uptake, and transport genes (such as *SREBP-1c*, *FAT/Cd36*, and *C/EBPα*) [8]. The effect of combined aerobic and resistance exercise on insulin sensitivity can be partially attributed to increased muscle tissue IRS-1 expression [21]. The present study extended these findings by proposing that combined aerobic and resistance exercise increased p-AMPK, SIRT1, and PPAR*α* expressions by downregulating GCN2 in WT mice. Given the essential role of AMPK in regulating hepatic lipid metabolism in response to nutritional challenges [22], SIRT1 was shown to be stimulated by AMPK activation via exercise, while lipolysis was found to be upregulated through increased fatty acid *β*-oxidation [23, 24]. Subsequently, exercise was noted to enhance the expressions of liver PPAR*α*, which played a crucial role in whole-body fatty acid homeostasis and protection against NAFLD [25, 26]. Thus, it was deemed plausible that combined aerobic and resistance exercise increased PPAR*α* activity through AMPK/SIRT1 pathways downstream of GCN2 inhibition, enhancing hepatocyte fat oxidation to guard against NAFLD.

The ability of combined aerobic and resistance exercise to produce liver lipid metabolic effects was seen to be enhanced in GCN2KO mice. A previous study showed that *GCN2* deficiency decreased the lipogenesis-related gene expressions (*PPARγ*, *FAS*, and *SREBP-1c*) in HFD-fed mice [15], suggesting that the loss of *GCN2* determined the increased proteins of key hepatic lipid metabolism either through direct translational regulation or indirectly through compensatory mechanisms. Based on the established physiological effects of PPAR*α* [27, 28], the present study indicated that exercise-induced decreases in liver weights and TG contents were consistent with the exercise-induced increase in hepatic PPAR*α*. This research also found that the exercise-dependent increase in PPAR*α* expression in the liver depended on AMPK/SIRT1 signaling in GCN2KO mice. These data suggested that besides the fact that GCN2-dependent activation of eIF2*α*/ATF4 was required for this signaling pathway, additional mechanisms including other upstream eIF2*α* kinases involved in controlling the adaptation of lipid metabolism in response to exercise were essential. Whether this pathway contributed to AMPK/SIRT1/PPAR*α* expressions in WT mice or was instead only recruited in the GCN2KO mice thus remains unclear.

Although hepatic PGC-1*α* is regarded as a powerfully detrimentally impacting blood glucose control by generating enzymes that drive gluconeogenesis [29, 30], research has shown that increased PGC-1*α* levels respond to insulin, counteracting uncontrolled glucose production through regulating the hepatic ratio of insulin receptor substrate 1 (IRS1) and IRS2 expression [31]. As one of the kinases of phosphorylating glycogen synthase (GS), GSK-3*β* is an essential factor leading to insulin resistance in the development of diabetes mellitus [32, 33]. Consistent with the functions of PGC-1*α* and GSK-3*β* in controlling glucose production as the hepatic response to insulin, the data from the present study proved that PGC-1*α* and p-GSK-3*β* were activated by exercise. Thus, the present work expanded the role of exercise in activating PGC-1*α* and in p-GSK-3*β* maintaining glucose homeostasis as a result of its effects on hepatic GCN2 inhibition. In addition to these findings regarding PGC-1*α* and GSK-3*β*, the study results identified that GLUT4 was activated by combined aerobic and resistance exercise. Although GLUT4 is not normally expressed in hepatocytes, it has been detected in hepatic stellate cells of mouse models and humans [34]. Reduced expression of hepatocyte GLUT4 in the liver of obesity-induced insulin resistance mouse models has been reported [35]. Study results have shown that the primary function of GLUT4 was to improve glucose tolerance in the liver of obese mice. Whether the GLUT4 signal comes from hepatocytes or stellate cells remains unclear, while interestingly, GLUT4 was found to have been upregulated by exercise. The present study findings suggested the remarkable possibility that GLUT4, PGC-1*α*, and p-GSK-3*β* activation following GCN2 exercise-induced inhibition could further increase hepatic response to insulin and improve glucose tolerance.

The present study further extended these findings in the exercised GCN2KO mice and suggested that GCN2 was generally required to regulate glucose metabolic response to exercise. Previous research by others had hypothesized that GCN2 may be a key molecular link regulator of insulin sensitivity and glucose metabolism [13, 36], while the reduced glucose tolerance in the exercised GCN2KO group may have resulted from a metabolic adaptation impacted by *GCN2* deficiency alongside a decreased response to insulin signaling. As shown and discussed above, exercise generated increased liver PGC-1*α*, GLUT4, and p-GSK-3*β* protein levels in the WT mice. These exercise-mediated inductions were markedly blunted in the GCN2KO group, and the lack of glucose metabolic responses to exercise in GCN2KO mice appeared to be explained by these blunt increases in PGC-1*α*, GLUT4, and p-GSK-3*β*. Interestingly, hepatic PGC-1*α* expression in the GCN2KO mice was significantly higher in WT mice, while the increase in PGC-1*α* may still have been insufficient to ensure blood glucose control in the GCN2KO group. Although hepatic PGC-1*α* contributed to controlling glucose production by regulating the IRS1 : IRS2 ratio, the effect of PGC-1*α* on increased blood glucose in GCN2KO mice remains unclear [31]. Additional research is required to define precisely the ratio of IRS1 and IRS2 expression in the liver of GCN2KO mice, and further studies are required to understand whether the impact of exercise on GCN2 loss in muscle tissue might be involved in the glucose-related alterations in the liver described above.

In summary, the present study findings provide evidence that combined aerobic and resistance exercise improved hepatic steatosis and glucose tolerance by downregulating GCN2 under HFD conditions. Using GCN2KO mice as an animal model, results revealed that exercise-induced improvement in glucose tolerance did require GCN2. Moreover, exercise can help prevent hepatic steatosis in the GCN2KO mice, suggesting that GCN2 may not be indispensable for exercise-induced hepatic steatosis improvement. Importantly, the present study indicates a possible mechanism whereby the combination of GCN2 deficiency and exercise may suppress the initiation of HFD-induced hepatic steatosis more effectively by activation of the AMPK/SIRT1/PPAR*α* pathway in the liver.

## Data Availability

The data generated and analysed during the current study are available from the corresponding author on reasonable request.
